# Pioneering strategies for overcoming bacterial drug resistance

**DOI:** 10.71150/jm.2603100

**Published:** 2026-03-31

**Authors:** Byoung Sik Kim

**Affiliations:** Department of Life Science, Sogang University, Seoul 04107, Republic of Korea

In the early 20th century, the introduction of antibiotics as "magic bullets"—agents capable of selectively targeting pathogens without harming the human body—led to an optimistic belief that humanity might finally overcome infectious diseases. However, their effectiveness has gradually diminished due to the global dissemination of antimicrobial resistance (AMR). Indeed, bacteria have continued to evolve over the past century. As a result, resistant strains have emerged that neutralize existing clinical options.

Unlike viral pandemics, antimicrobial resistance (AMR) is a “silent pandemic” that advances slowly yet poses a persistent threat. As a result, its danger is often underestimated. Recent data reveal that in 2019 alone, 1.27 million deaths were directly caused by AMR, with another 4.95 million associated with it (Murray et al., 2022). The growing multidrug resistance of high-risk pathogens, collectively known as the ESKAPE group, has become increasingly severe, and the COVID-19 pandemic has further intensified this trend by driving the excessive use of prophylactic antibiotics (Langford et al., 2023).

Despite this crisis, the approval of new antibiotic classes has remained scarce over the past two decades, largely because major pharmaceutical companies have halted development owing to high R&D costs and low profit margins. Since conventional single-target small molecules make it easier for bacteria to evolve resistance, innovative approaches with entirely new mechanisms are urgently required. In this Special Issue, seven review articles highlighting next-generation antimicrobial strategies, including hybrid antibiotics, peptide-based agents, β-lactamase inhibitors, proteostasis-targeting strategies, and synthetic biology-driven solutions are presented.

## Advancements of Hybrids and Combination Therapies

To address the global AMR crisis, innovative design strategies that move beyond the traditional single-drug paradigm are required. In this context, Lee et al. (2026) provide an in-depth analysis of antibiotic hybrids, a strategy that combines two antibiotics or functional modules into a single compound. Their review shows that hybrid antibiotics, used as single agents, can simplify complex pharmacokinetic/pharmacodynamic (PK/PD) analyses and dosing regimens. In addition, this approach can improve the efficiency of drug development by using clinically validated drugs as building blocks. The authors describe recent breakthroughs, such as “Trojan horse” strategies using β-lactam-based siderophore conjugates, and efforts to extend the spectrum of Gram-positive-only antibiotics, like vancomycin and daptomycin, to Gram-negative pathogens. By examining key examples such as Cefiderocol and several Phase 3 candidates (e.g., TNP-2092, TNP-2198, and TD-1792), the review argues that precise optimization of linker technology will be essential for closing critical gaps in the current antibiotic pipeline.

Consistently, Son et al. (2026) extend the discussion by focusing on recent trends in dual-acting hybrid antibiotics and combination therapies specifically designed for Gram-negative pathogens. Their review emphasizes that hybrid antibiotics can bypass bacterial efflux mechanisms using metabolically stable covalent bonds. The authors discuss various hybrid antibiotics that combine quinolones with cephalosporins and aminoglycosides, alongside several clinical candidates (e.g., fluoroquinolone-oxazolidinone hybrids). However, they caution that efficacy against Gram-negative pathogens remains uncertain due to limited in vivo studies and the primary focus of clinical trials on Gram-positive bacteria. They also note a key physical limitation, namely that high molecular weight can hinder penetration of the Gram-negative outer membrane. To overcome this, the authors recommend optimizing physicochemical properties through strategies like siderophore conjugation and adjustments to charge distribution and amphiphilicity.

## Targeted Inhibitors and Proteostasis Management

Another key approach to preserving antibiotic efficacy is to neutralize bacterial defense mechanisms and target systemic vulnerabilities. Park et al. (2026) provide a comprehensive review of the structural and mechanistic features of β-lactamases and their inhibitors (BLIs), which are used to protect β-lactam antibiotics that account for more than half of global antibiotic consumption. From a structural biology perspective, the review describes how eight FDA-approved inhibitors bind to enzyme active sites according to their chemical scaffolds [β-lactams, diazabicyclooctanes (DBOs), or boronic acids]. Notably, it discusses the unique recycling mechanism of DBO-based inhibitors and the potential of boronic acid scaffolds to act as pan-class inhibitors targeting both serine and metallo-β-lactamases, despite the current FDA-approved Vaborbactam being limited to serine β-lactamases. Although some resistance-conferring mutations are not yet fully addressed in clinical settings, the review offers important guidance for designing next-generation universal inhibitors by clarifying the structural basis of these variants.

Beyond blocking specific enzymatic functions, strategies that disrupt the networks maintaining overall cellular integrity are also gaining attention. Jeong et al. (2026) examine next-generation antimicrobial strategies that target the protein quality control (PQC) system, a key determinant of bacterial survival and resistance. Their review focuses on innovative approaches that modulate molecular chaperones (DnaK, GroEL/ES, ClpB) or AAA+ proteases (ClpP, Lon) either by inhibiting or enhancing their functions. Strikingly, technologies such as aggregation-prone peptides (APPs), which induce widespread protein aggregation, and bacterial PROTACs (BacPROTACs), which direct endogenous proteases to specific target proteins, have shown strong bactericidal effects even in persisters and VBNC (viable but non-culturable) models. These findings support the idea that inducing a systemic collapse of proteostasis can serve as a powerful antimicrobial strategy.

## Next-Generation Peptide-Based Antimicrobials

As a new modality, peptides are considered as one of the most promising alternatives because they offer substantial design flexibility. Kim (2026) systematically organizes recent advances and future prospects of peptide-based antibiotics according to their origin, structure, and mode of action. The review highlights artificial intelligence (AI)-assisted design, production optimization via synthetic biology, and improved in vivo stability through drug delivery systems as key areas of innovation. By assessing the commercialization potential of agents ranging from established drugs like daptomycin to clinical candidates such as PLG0206 and murepavadin, the author argues that the convergence of AI, synthetic biology, and advanced delivery technologies is likely to become a major driver in addressing public health crises.

These peptide strategies are also being extended to the treatment of fungal infections. Kim et al. (2026) offer a novel perspective by targeting ribosome homeostasis through peptide-based modulation to overcome the limitations of conventional antifungals. To address the challenge posed by the high structural conservation between fungal and human ribosomes, the authors focus on ribosome-associated proteins (e.g., Tsr4, Tsr2, Atg11, Stm1) that show relatively low sequence conservation. Their precision strategy, which uses short peptides derived from ribosomal proteins to competitively block these interaction interfaces and disrupt fungal proteostasis, is proposed as a promising conceptual framework for novel antifungals, though the authors note that empirical testing and delivery optimizations are still required.

## Synthetic Biology and Convergent Technologies

A final key area involves engineering platforms to accelerate and refine these antimicrobial strategies. Oh et al. (2026) discuss how synthetic biology is reshaping the traditional antibiotic paradigm. Their review covers four major approaches: selective removal of resistance genes using CRISPR-Cas systems, engineered bacteriophages with expanded host ranges, microbiome engineering using live biotherapeutics and in situ genetic modification to suppress resistant pathogens and gene transfer, and discovery of novel antimicrobials through metabolic engineering. These technologies are expected to play a crucial role not only in killing resistant bacteria but also in developing sustainable resistance management strategies and precision medicine approaches.

## Concluding Remarks and Future Perspective

Collectively, the studies in this Special Issue showcase diverse strategies to overcome the limitations of conventional antibiotic development. Peptide-based therapies offer high target selectivity and novel mechanisms of action that reduce resistance potential, while advances in AI-driven design and manufacturing are bringing them closer to clinical reality. Similarly, dual-acting hybrid antibiotics and novel β-lactamase inhibitors promise to extend the utility of our existing drug arsenal.

Moving forward, integrating AI, precision drug delivery systems, and synthetic biology will drive AMR innovation. Approaches that disrupt bacterial proteostasis to eliminate persister cells, or use CRISPR-Cas and engineered phages to target resistance genes directly, point toward a new era in antimicrobial therapy.

However, translating these advances into clinical practice will require addressing biological complexity and industrial stability challenges. Ethical and regulatory issues must also be carefully considered. Ultimately, only through close collaboration among researchers, industry, and policymakers can we develop sustainable solutions to this silent crisis. Finally, I am grateful to all authors for their outstanding contributions and hope this Special Issue inspires continued efforts to combat antimicrobial resistance.
